# Association between carotid plaque vulnerability and high mobility group box-1 serum levels in a diabetic population

**DOI:** 10.1186/s12933-021-01304-8

**Published:** 2021-05-27

**Authors:** Federico Biscetti, Giovanni Tinelli, Maria Margherita Rando, Elisabetta Nardella, Andrea Leonardo Cecchini, Flavia Angelini, Giuseppe Straface, Marco Filipponi, Vincenzo Arena, Dario Pitocco, Antonio Gasbarrini, Massimo Massetti, Andrea Flex

**Affiliations:** 1 Fondazione Policlinico Universitario A. Gemelli IRCCS, Roma, Italy; 2 Cardiovascular Internal Medicine, Fondazione Policlinico Universitario A. Gemelli IRCCS, Catholic University School of Medicine, Largo Francesco Vito, 1, 00168 Roma, Italy; 3 Laboratory of Vascular Biology and Genetics, Università Cattolica del Sacro Cuore, Roma, Italy; 4 Vascular Surgery, Fondazione Policlinico Universitario A. Gemelli IRCCS, Roma, Italy; 5 Università Cattolica del Sacro Cuore, Roma, Italy; 6 Department of Internal Medicine, St. M. Goretti Hospital, Roma, Italy; 7 Ospedale San Giovanni Battista-ACISMOM, Roma, Italy; 8 Department of Pathology, Fondazione Policlinico Universitario A. Gemelli IRCCS, Roma, Italy; 9 Diabetology Unit, Fondazione Policlinico Universitario A. Gemelli IRCCS, Roma, Italy; 10 Department of Internal Medicine, Fondazione Policlinico Universitario A. Gemelli IRCCS, Roma, Italy; 11 Cardiovascular Surgery, Fondazione Policlinico Universitario A. Gemelli IRCCS, Roma, Italy

**Keywords:** Diabetes mellitus, Internal carotid artery stenosis (ICAS), High mobility group box-1 (HMGB1)

## Abstract

**Background:**

Carotid atherosclerosis represents one of the complications of diabetes mellitus. In particular, plaque instability contributes to disease progression and stroke incidence. High mobility group box-1 (HMGB1) is a nuclear protein involved in promotion and progression of atherosclerosis and cardiovascular diseases. The aim of this study was to analyze the relationship between HMGB1 serum levels, main inflammatory cytokines, the presence of internal carotid stenosis and unstable plaque in a diabetic population.

**Research design and methods:**

We studied 873 diabetic patients, including 347 patients with internal carotid artery stenosis (ICAS) who underwent carotid endarterectomy and 526 diabetic patients without internal carotid artery stenosis (WICAS). At baseline, HMGB1 and the main inflammatory cytokines serum levels were evaluated. For ICAS patients, the histological features of carotid plaque were also collected to differentiate them in patients with stable or unstable atherosclerotic lesions.

**Results:**

We found that HMGB1 serum levels, osteoprotegerin, high-sensitivity C-reactive protein, tumor necrosis factor-alpha and interleukin-6, were significantly higher in diabetic ICAS patients compared to diabetic WICAS patients. Among ICAS patients, individuals with unstable plaque had higher levels of these cytokines, compared to patients with stable plaque. A multivariable stepwise logistic regression analysis showed that HMGB1 and osteoprotegerin remained independently associated with unstable plaque in ICAS patients.

**Conclusions:**

The present study demonstrated that HMGB1 is an independent risk factor for carotid plaque vulnerability in an Italian population with diabetes mellitus, representing a promising biomarker of carotid plaque instability and a possible molecular target to treat unstable carotid plaques and to prevent stroke.

## Background

Cerebrovascular disease (CVD) represents a major cause of disability and mortality, with 5.5 million deaths due to stroke worldwide [1]. In particular, carotid atherosclerosis is responsible for 15% of ischemic strokes [2], and unstable carotid plaques correlate with a major risk of cerebrovascular events and stroke recurrence [3, 4]. Systemic inflammation plays a pivotal role on plaque development and vulnerability [5, 6] and several studies have analyzed the linkage between carotid atherosclerosis and inflammatory biomarkers, such as high-sensitivity C-reactive protein (Hs-CRP), interleukin (IL)-6, serum amyloid A, fibrinogen, lipoprotein-associated phospholipase A2 (Lp-PLA2) [6–12].

High mobility group box-1 (HMGB1) is a DNA-binding protein, which acts in the extracellular space as a pro-inflammatory cytokine [13], promoting inflammatory response, tissue regeneration and angiogenesis [14, 15]. HMGB1 can be passively released by necrotic cells or actively secreted by immune cells, after different stimuli, such as oxidative stress and cytokines release, as IL-6, IL-1, tumor necrosis factor-alpha, interferon-gamma [14, 16]. In the extracellular milieu, HMGB1 acts by linking to the toll-like receptors and advanced glycation end product receptors, enhancing the inflammatory response, chemotaxis, immune and endothelial cells activation and differentiation [14].

HMGB1 also promotes atherosclerosis progression with a significant role in cardiovascular disease development [17–20]. Interesting data suggests that in ApoE-/-mice, monoclonal anti-HMGB1 neutralizing antibodies reduced atherosclerosis, macrophages, smooth muscle and dendritic cell accumulations, and they reduced vascular cell adhesion molecule-1 and monocyte chemoattractant protein-1 expression [21]. Further reports showed that the number of macrophages with expression of nuclear and cytoplasmic HMGB1 is elevated in fatty streaks and fibro-fatty lesions of thoracic and abdominal aorta samples [16], contributing to inflammation of atherosclerotic fatty plaques [16]. It has been also shown that HMGB1 is involved in non-calcified coronary plaques and remodeled plaques in patients with stable coronary artery disease [22]. Moreover, HMGB1 levels correlate with the severity of coronary artery disease [23]. HMGB1 plays an important role even in peripheral arterial disease and its severity; in fact, diabetic patients with limb ischemia had higher HMGB1 serum levels with respect to those with stable peripheral arterial disease [24]. Furthermore, in a diabetic population, higher levels of HMGB1 and osteoprotegerin, a member of the tumor necrosis factor (TNF) receptor superfamily, have been associated to peripheral arterial disease [24]. In addition, an increased HMGB1 expression in vascular smooth muscle cells (VSMCs) of carotid and coronary atherosclerotic lesions has been reported [25]. However, there are no evidences about HMGB1 serum levels and the role on carotid plaque vulnerability.

Given available data, we hypothesize that HMGB1 might be associated to carotid plaque vulnerability. The principal aim of this study is to evaluate the relationship between HMGB1 serum levels and carotid plaque stability in a diabetic population of patients with internal carotid artery stenosis (ICAS) in need of carotid endarterectomy, compared to diabetic patients without ICAS (WICAS). An additional purpose is to assess levels of main inflammatory cytokines involved in atherosclerosis of these patients.

## Methods

### Study population

A total of 873 consecutive Caucasian patients with type 2 diabetes mellitus (T2DM) were enrolled from June 1, 2018, to February 15, 2021. Specifically, we included 347 diabetic patients with ICAS who underwent carotid endarterectomy admitted to the Departments of Vascular Surgery of the Fondazione Policlinico Universitario A. Gemelli IRCCS, Roma, Italy, and the Santa Maria Goretti Hospital, Latina, Italy. Additional 526 diabetic WICAS patients, admitted to the Department of Vascular Medicine of the Fondazione Policlinico Universitario A. Gemelli IRCCS, Roma, Italy were included in the study.

At time of enrollment, all diabetic patients underwent an interview to collect data about clinical history and presence of cardiovascular risk factors, in particular age, hypertension, hypercholesterolemia and smoking habits. They underwent also a detailed clinical examination, an ECG at rest, an ultrasound of carotid arteries and cerebral CT-scan to assess the presence of acute cerebrovascular disease, such as cerebral infarction. The diagnosis of T2DM was confirmed according to previous studies [26–31]. Blood hypertension was defined as a systolic blood pressure  ≥  130 mmHg and a diastolic blood pressure  ≥  85 mmHg or presence of active anti-hypertensive treatment. Hypercholesterolemia was defined as a serum cholesterol level  ≥  220 mg/dL or presence of active lipid-lowering drugs. A person with any amount of smoke per day was defined as current smoker. A person who had stopped smoking more than a year before the study was defined as former smoker. Height and weight were measured and expressed as body mass index (BMI), defined as weight/height^2^ (kg/m^2^). As previously described, a carotid endarterectomy was performed according to standardized criteria [26] and a ultrasound evaluation of plaque density by color-coded echo flow imaging confirmed by angiography was performed before the procedure [26]. Exclusion criteria were pregnancy, active cancer, life expectancy  < 6 months, known liver disease with a functional status according to Child–Pugh classification of B or above, congenital or acquired thrombophilia and active autoimmune disease.

This study was approved by the ethics committee of the Fondazione Policlinico Universitario A. Gemelli IRCCS, Roma, Italy and St. M. Goretti Hospital, Latina, Italy. All the patients included gave the informed consent to the enrollment.

### Histological assay

**Fig. 1:**
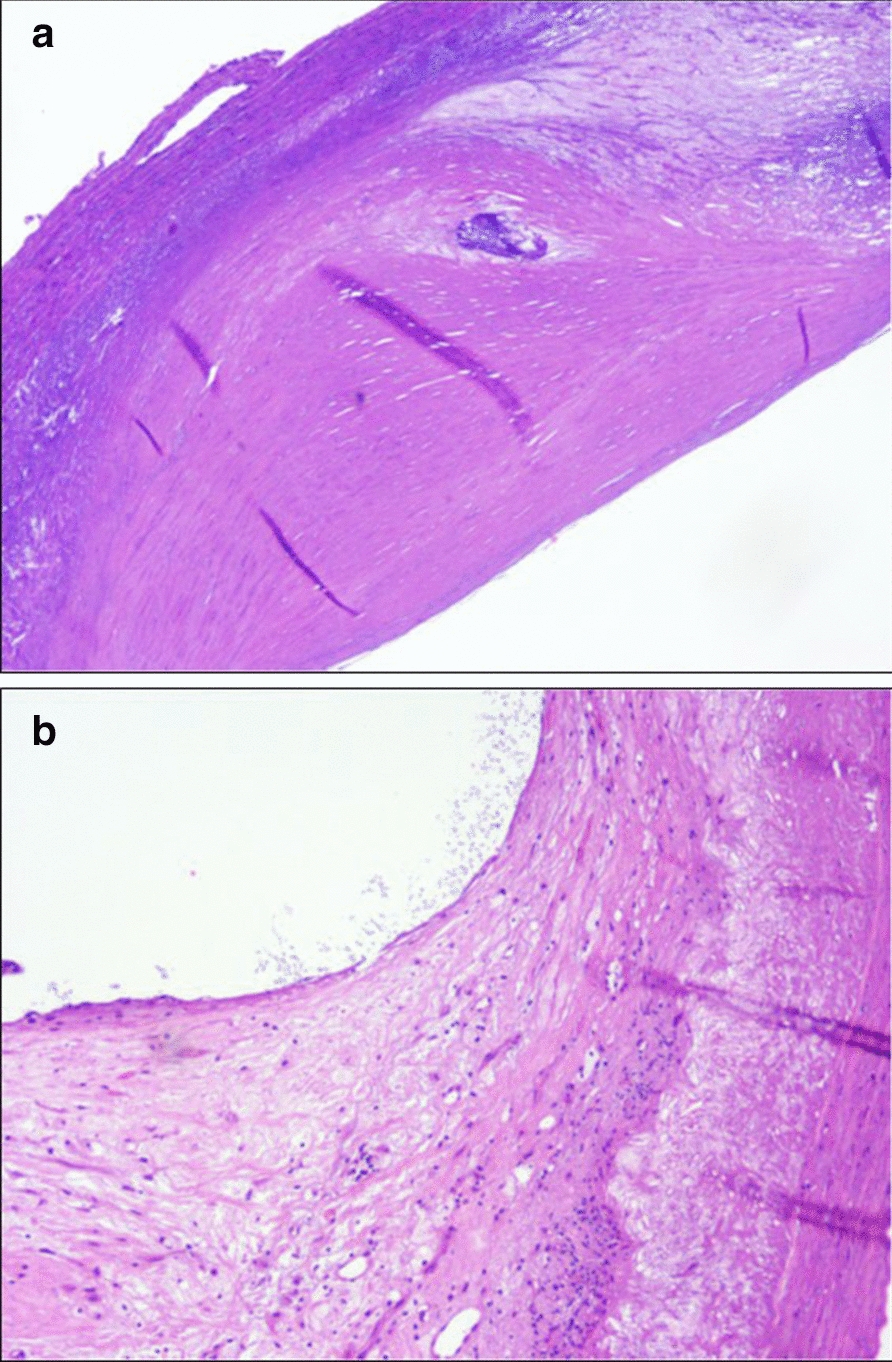
**A** Fibrous cap atheroma (stable plaque). **B** Healed plaque with multilayering of collagen and a paucity of smooth muscle cells (unstable plaque). Hematoxylin and eosin stain 20 ×

The carotid endarterectomy was performed as previously described [26]. After the surgery the specimens were briefly rinsed in normal saline solution and then immersed in a buffered 10% formalin fixative and, subsequently, in a decalcifying solution (formic acid). The plaques were partly decalcified to be sectioned. Each specimen was sectioned transversely, perpendicular to the lumen, into 4 mm blocks, starting from the specimen base, and then, progressing distally until the whole specimen, including the bifurcation, was cut. Each block was processed in paraffin, cut into 4 μm sections, and then, the proximal end of one slice per block was stained in sequence with hematoxylin and eosin 1%. The sections were analyzed to evaluate the presence of atheroma, a necrotic core, hemorrhages, fibrosis, calcifications and thrombosis. Minimum magnification was used to examine the relative necrotic core, and maximum magnification was used to assess the presence of hemorrhages and calcifications. The morphological study was conducted on the largest plaque area, corresponding to the level of maximal stenosis. The necrotic core was made of cholesterol clefts and amorphous material devoid of viable cells or admixed collagen, the deepest portion of the atherosclerotic plaque. In the hematoxylin and eosin stain calcifications appeared as dark blue, sharply demarcated regions without cells. Intra-plaque hemorrhages appeared as debris of degenerated erythrocytes, with macrophage engulfment of hemosiderin and giant cells. Carotid plaques were classified as stable or unstable (Fig. 1) according to the American Heart Association (AHA) criteria [32], later redefined by Virmani and et al. [33].

### Biochemical investigation

For each patient involved in the study, blood samples were collected and white blood cell count, serum creatinine, fasting cholesterol, triglycerides and low-density lipoprotein were determined after an overnight fast. Serum was separated by centrifugation of blood samples and stored at  − 80 °C, before measurement. Serum HMGB1 level had been determined by a commercially available ELISA kit (HMGB1 ELISA kit II; Shino-Test Corporation, Tokyo) according to its protocol. The detection limit for HMGB1 was 0.2 ng/mL with an inter-assay coefficient of variation  <  10% [24]. Hs-CRP levels were assessed with a high-sensitivity ELISA kit (Biocheck Laboratories, Toledo, OH, USA). Serum IL-6 and TNF-alpha levels were determined through the Quantikine ELISA kit (R&D Systems, Minneapolis, MN, USA). A monoclonal mouse antihuman osteoprotegerin antibody was used as a capture antibody and a biotinylated polyclonal goat antihuman osteoprotegerin antibody (R&D systems) was used for detection. The intra and inter-assay coefficients of variation were 3.6 and 10.6%, respectively. The sensitivity, defined as the mean  ±  3 SD of the 0 standard, was calculated to be 0.15 pmol/mL. For all participants, the serum levels were measured twice and the results were averaged.

### Statistical analysis

Demographic and clinical data were compared with Chi squared test and Student’s t test between the two groups. HMGB1, osteoprotegerin, Hs-CRP, IL-6 and TNF-alpha levels were compared using the Mann–Whitney test. Two models were tested using a multivariate stepwise logistic regression analysis. The first one was adjusted for traditional risk factors, while HMGB1, osteoprotegerin, Hs-CRP, IL-6 and TNF-alpha were included for testing in the second model. All analyses were performed using STATA version 11.0 for Windows (Statistics/Data Analysis, Stata Corporation, College Station, TX, USA). Statistical significance has been established at p  <  0.05.

## Results

The demographic and clinical data of diabetic patients with ICAS and WICAS are summarized in Table 1. There were no significant differences between the groups in terms of sex (p  =  0.384), age (p  =  0.157), diabetes duration (p  =  0.209), current and former smoking (p  =  0.185 and p  =  0.261, respectively).

**Table 1:** Demographic and clinical data of study participants

	ICAS (n = 347)	WICAS (n = 526)	p value
Men/female, n	118:229	192:334	0.384^b^
Age (years ± SD)	72.1 ± 3.9	71.8 ± 3.8	0.157^a^
Diabetes duration, (years ± SD)	13.3 ± 4.5	12.2 ± 3.4	0.209^a^
BMI (Kg/m2 ± SD)	30.7 ± 6.3	27.9 ± 4.6	< 0.01^a^
Smoking (current), n (%)	116 (33.4)	172 (32.7)	0.185^b^
Smoking (former), n (%)	145 (41.8)	221 (42.0)	0.261^b^
Hypertension, n (%)	221 (63.7)	215 (40.9)	< 0.01^b^
CAD, n (%)	189 (54.5)	164 (31.2)	< 0.01^b^
HIS, n (%)	164 (47.3)	0 (0.0)	< 0.01^b^
PAD, n (%)	119 (34.3)	113 (21.5)	< 0.01^b^
Hypercholesterolemia, n (%)	275 (79.3)	153 (29.1)	< 0.01^b^
LDL-C (mg/dL ± SD)	154 ± 14.6	102 ± 11.2	< 0.01^b^
Triglycerides (mg/dL ± SD)	194 ± 20.1	139 ± 15.4	< 0.01^b^
Statins, n (%)	274 (78.9)	156 (29.7%)	< 0.01^b^
Antihypertensive drugs, n (%)	223 (64.3)	214 (40.7)	< 0.01^b^
Anti-diabetic treatment			
Diet only, n (%)	36 (10.4)	56 (10.6)	0.349^b^
Oral agents, n (%)	169 (48.7)	259 (49.2)	0.328^b^
Insulin therapy, n (%)	142 (40.9)	211 (40.2)	0.243^b^

*ICAS* internal carotid artery stenosis; *WICAS* without internal carotid artery stenosis; *BMI* body mass index; *CAD* coronary artery disease; *HIS* history of ischemic stroke; *PAD* peripheral artery disease; *LDL-C* low-density lipoprotein cholesterol

^a^Statistical test performed with Student’s t test

^b^Chi-square test for categorical values

Respect to diabetic WICAS patients, diabetic patients with ICAS had higher values of BMI (p  <  0.01), blood pressure (p  <  0.01) and hypercholesterolemia (p  <  0.01). Moreover, coronary artery disease (p  <  0.01), history of ischemic stroke (p  <  0.01) and peripheral artery disease (p  <  0.01) were more frequent in the ICAS group (Table 1). Among diabetic patients with ICAS, 36 (10.4%) patients were treated with diet only, 169 (48.7%) were treated with oral agents and 142 (40.9%) were treated with insulin and there were no differences in the two groups analyzed, in term of diabetic treatments.

Median serum levels of HMGB1, osteoprotegerin, Hs-CRP, TNF-alpha and IL-6, were significantly higher in diabetic ICAS patients respect to diabetic WICAS patients (Table 2). In particular, the median serum HMGB1 was 2.96 (± 7.45) ng/mL in WICAS and 7.65 (± 13.32) ng/mL in ICAS (p  <  0.001) patients; the median serum osteoprotegerin was 3.23 (± 2.25) pmol/L in WICAS and 6.86 (± 6.55) pmol/L in ICAS (p  <  0.001) patients; the median serum Hs-CRP was 7.92 (± 3.32) mg/L in WICAS and 16.7 (± 13.2) mg/L in ICAS (p  <  0.001) patients; the median serum IL-6 was 34.3 (± 14.5) pg/mL in WICAS and 52.4 (± 16.3) pg/ mL in ICAS (p  <  0.001) patients (Table 2). The median serum TNF-alpha was 58.3 (± 21.2) pg/mL in WICAS and 63 (± 32.2) in ICAS patients, but the differences between the groups were not statistically significant (p  =  0.384) (Table 2).

**Table 2:** Serum levels in diabetic patients with and without ICAS

Variables	ICAS (n = 347)	WICAS (n = 526)	p value
HMGB1 (ng/mL ± SD)	7.65 ± 13.32	2.96 ± 7.45	< 0.001^a^
OPG (pmol/L ± SD)	6.86 ± 6.55	3.23 ± 2.25	< 0.001^a^
Hs-CRP (mg/L ± SD)	16.7 ± 13.2	7.92 ± 3.32	< 0.001^a^
TNF-alpha (pg/mL ± SD)	63.0 ± 32.2	58.3 ± 21.2	< 0.384^a^
IL-6 (pg/mL ± SD)	52.4 ± 16.3	34.3 ± 14.5	< 0.001^a^

*ICAS* internal carotid artery stenosis; *WICAS* without internal carotid artery stenosis; *SD* standard deviation; *HMGB1* high-mobility group box 1; *OPG* osteoprotegerin; *Hs-CRP* high-sensitivity C-reactive protein; *TNF-alpha* tumor necrosis factor-alpha; *IL-6* interleukin-6

^a^ Statistical test performed with Student’s t test

Afterward, the 347 diabetic patients with ICAS were divided in two different groups: unstable plaque group (n  =  159) and stable plaque group (n  =  188). Demographic and clinical data of these groups are summarized in Table 3. Patients with unstable plaque had more frequently a history of ischemic stroke (HIS) (p  =  0.001), hypercholesterolemia (p  =  0.003) and they had higher values of low-density lipoprotein cholesterol (LDL-C) (p  =  0.001), compared to stable plaque patients (Table 3). Serum levels of HMGB1, osteoprotegerin, Hs-CRP, TNF-alpha and IL-6 were subsequently analyzed in these two groups as shown in Table 4. Interestingly, unstable plaque patients had higher levels of these cytokines. In particular, the median serum HMGB1 was 8.19 (± 11.34) ng/mL in unstable plaque and 4.35 (± 6.49) ng/mL in stable plaque (p  <  0.001); the median serum osteoprotegerin was 7.65 (± 8.12) pmol/L in unstable plaque and 3.13 (± 2.23) pmol/L in stable plaque (p  <  0.001); the median serum Hs-CRP was 17.3 (± 11.2) mg/L in unstable plaque and 8.9 (± 4.3) mg/L in stable plaque (p  <  0.001); the median serum IL-6 was 54.2 (± 15.2) pg/mL in unstable plaque and 27.2 (± 9.2) pg/ mL in stable plaque controls (p  <  0.001) (Table 4).

**Table 3:** Demographic and clinical data of ICAS patients with stable and unstable plaque

	USP (n = 159)	SP (n = 188)	p value
Men/female, n	57:102	69:119	0.321^b^
Age (years ± SD)	72.2 ± 3.3	72.3 ± 3.9	0.241^a^
Smoking (current), n (%)	53 (33.3)	63 (33.5)	0.157^b^
Smoking (former), n (%)	65 (40.9)	80 (42.4)	0.159^b^
Hypertension, n (%)	102 (64.2)	119 (63.3)	0.214^b^
CAD, n (%)	86 (54.1)	103 (54.8)	0.181^b^
HIS, n (%)	98 (61.6)	66 (35.1)	0.001^b^
PAD, n (%)	55 (34.6)	64 (34.0)	0.237^b^
Hypercholesterolemia, n (%)	133 (83.6)	142 (75.5)	0.003^b^
LDL-C (mg/dL ± SD)	177 ± 24.2	132 ± 18.2	0.001^b^

*USP* unstable plaque; *SP* stable plaque; *CAD* coronary artery disease; *HIS* history of ischemic stroke; *PAD* peripheral artery disease; *LDL-C* low-density lipoprotein cholesterol

^a^Statistical test performed with Student’s t test

^b^Chi-square test for categorical values

**Table 4:** Serum levels in diabetic patients with stable and unstable plaques

	USP (n = 159)	SP (n = 188)	p value
HMGB1 (ng/mL ± SD)	8.19 ± 11.34	4.35 ± 6.49	< 0.001^a^
OPG (pmol/L ± SD)	7.65 ± 8.12	3.13 ± 2.23	< 0.001^a^
Hs-CRP (mg/L ± SD)	17.3 ± 11.2	8.9 ± 4.3	< 0.001^a^
IL-6 (pg/mL ± SD)	54.2 ± 15.2	27.2 ± 9.2	< 0.001^a^

*SD* standard deviation; *HMGB1* high-mobility group box 1; *OPG* osteoprotegerin; *Hs-CRP* high-sensitivity C-reactive protein; *IL-6* interleukin-6

^a^ Statistical test performed with Student’s t test

After adjustment for traditional cardiovascular risk factors and established inflammatory cytokines, a multivariable stepwise logistic regression analysis, in model 1, showed that sex, age, smoking, hypertension, hypercholesterolemia, triglycerides, LDL-C, Hs-CRP and IL-6 levels were independent determinants of unstable plaque in diabetic patients with ICAS. Including HMGB1 and osteoprotegerin in the multivariable analysis in model 2, HMGB1 and osteoprotegerin remained independently associated with unstable plaque in ICAS patients, and the conventional risk factors in model 1 remained determinants of unstable plaque even in model 2 (Table 5).

**Table 5:** Multivariable stepwise logistic regression model for presence of unstable plaques

	Variable OR (95% CI)	p value
Model 1		
Sex	1.12 (0.95–1.14)	0.206
Age	1.24 (1.09–1.32)	0.182
Smoking (current)	22.3 (3.98–87.1)	0.001
Smoking (former)	1.44 (1.12–1.53)	0.185
Hypertension	21.1 (5.45–64.1)	< 0.001
Hypercholesterolemia	7.21 (2.34–17.9)	0.021
Triglycerides	0.64 (0.23–1.24)	0.041
LDL-C	9.31 (1.62–53.1)	0.023
Hs-CRP	29.5 (9.08–86.4)	< 0.001
IL-6	6.76 (2.37–15.8)	0.001
Model 2		
Sex	0.89 (0.20–0.97)	0.096
Age	1.33 (1.04–1.72)	0.216
Smoking (current)	46.4 (4.24–165.5)	0.001
Smoking (former)	1.34 (1.09–1.57)	0.212
Hypertension	29.3 (4.87–143.3)	< 0.001
Hypercholesterolemia	8.34 (2.06–39.6)	0.011
Triglycerides	0.91 (0.45–1.79)	0.003
LDL-C	21.3 (3.58–149.5)	0.012
Hs-CRP	65.4 (9.33–196.3)	< 0.001
IL-6	7.69 (2.92–18.91)	< 0.001
OPG	4.34 (1.98–5.89)	0.021
HMGB1	19.6 (4.75–27.39)	0.015

Model 1: adjusted for traditional cardiovascular risk factors and established inflammatory cytokines

Model 2: adjusted for the risk factors in model 1 plus high-mobility group box 1 (HMGB1) and osteoprotegerin (OPG)

## Discussion

In this study, we demonstrated that serum levels of HMGB1 are independently associated with unstable carotid plaque in T2DM patients. In particular, we found that levels of HMGB1 are significantly higher in diabetic patients with ICAS and unstable plaque, respect to diabetic patients with ICAS and stable plaque (8.19 ± 11.34 ng/mL and 4.35 ± 6.49 ng/mL, p  <  0.001, respectively); moreover, ICAS patients with unstable plaque showed more frequently a history of stroke (61.6%, p  <  0.001). As shown in our previous report, even levels of osteoprotegerin, Hs-CRP and IL-6 are correlated significantly with unstable carotid plaque [26]. Moreover, in this study, a positive correlation regarding levels of these cytokines in ICAS patients was found. In fact, serum levels of HMGB1, osteoprotegerin, Hs-CRP, IL-6 were higher in diabetic ICAS patients when compared to WICAS controls. The multivariable stepwise logistic regression analysis showed also that HMGB1 and osteoprotegerin remained independently associated in diabetic patients with ICAS and unstable plaque (model 2) after adjustment for conventional cardiovascular risk factors.

These findings are in line with several evidences that demonstrated a role of HMGB1 in atherosclerosis and in vascular complications of T2DM [14, 34, 35]. In particular, HMGB1 is a ubiquitous protein, which acts, in the extracellular space, as a pro-inflammatory factor [36], binding advanced glycation end product receptors and toll-like receptors. Inflammation is a determinant factor in promoting atherosclerosis [37]. Moreover, inflammation, proteolysis and reduced collagen content promote rupture of atherosclerotic plaques [38]. HMGB1 has recently been recognized as a determining factor in atherosclerosis formation and development [39]. Porto and colleagues demonstrated that, respect to normal vessels, carotid atherosclerotic lesions contain large amount of HMGB1, which is actively secreted by monocytes, macrophages, dendritic cells and VSMCs. Moreover, they showed that an atherogenic stimulus as cholesterol could enhance secretion of HMGB1 from VSMCs in vitro. HMGB1 promotes even VSMCs replication, which can justify neointima hyperplasia during atherosclerosis development and restenosis after coronary angioplasty [40]. In fact, after plaque rupture, VSMCs accumulate at this site and, through secretion of extracellular matrix rich in glycosaminoglycans and type III collagen; they enhance plaque progression and constrictive remodeling of the arterial wall [41]. Furthermore, inhibition of HMGB1 activity suppressed VSMCs replication and it inhibited neointimal formation after carotid injury in animals’ model [42]. Moreover, cilostazol, a drug commonly used in peripheral arterial disease, is able to inhibit the secretion of HMGB1 [43] and to reduce the volume of carotid atherosclerotic plaque [44, 45]. Another study demonstrated that coronary and carotid atherosclerotic arteries showed high expression of HMGB1 in VSMCs. These cells expressed high levels of advanced glycation end product receptors, IL-6 and CRP too [25]. These cytokines, including HMGB1, seem to promote matrix metalloproteinase (MMP) secretion by VSMCs. In particular, HMGB1 stimulates MMP-2 mRNA expression by cultured VSMCs [25]. As known, MMPs play a key role in matrix degradation, contributing to vulnerability of atherosclerotic plaque [25]. The evidences discussed are in line with the findings of our study. In fact, we demonstrate that high levels of HMGB1 are associated with the presence of unstable plaque and a more frequent history of stroke. Therefore, HMGB1 could play a key role in plaque vulnerability and it could represent a predictor marker of plaque rupture.

Finally, alongside the role of HMGB1, our study confirms the importance of inflammation in plaque instability and tendency to rupture, already demonstrated in other patient cohorts [46]. When it was discovered, HMGB1 appeared to be part of an ancestral system of sterile inflammation [47]. Over the years it has been shown that this nuclear protein, when actively secreted, can activate a series of inflammatory mechanisms crucial in the development of atherosclerotic plaque [14, 48]. We found that osteoprotegerin, Hs-CRP, TNF-alpha and IL-6 serum levels, were significantly higher in diabetic ICAS patients respect to diabetic WICAS patients, suggesting the presence of an “inflammatory signature” in our patient cohort.

**Fig. 2:**
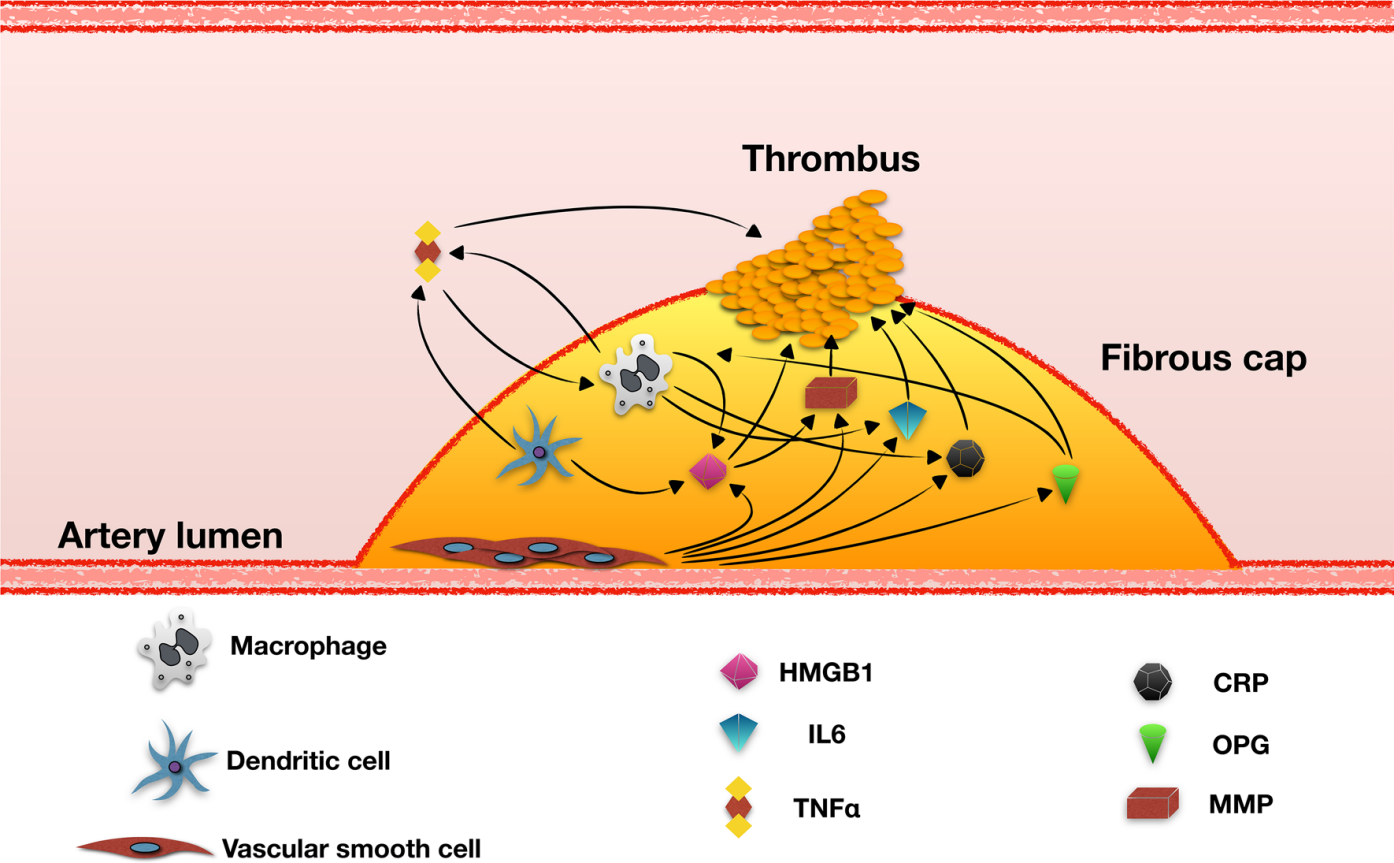
Schematic representation of the relationships between the different cells and cytokines involved in the instability of atherosclerotic plaque

Our evidence allows us to hypothesize a higher risk profile for ICAS patients with elevated plasma HMGB1, osteoprotegerin, Hs-CRP, TNF-alpha and IL-6 serum levels (Fig. 2). Together with the traditional risk factors for atherosclerosis, such as smoking, diabetes, hypertension and dyslipidemia, we can consider these inflammatory pathways as possible biomarkers of risk of more aggressive disease. Additional data to define the risk of the patient with ICAS could be useful in determining the most appropriate therapeutic approach and a truly personalized follow-up.

There are several limitations in this study. In fact, it is a case-control study, with possible survival and enrollment bias. Moreover, the cohort represented is based on an Italian population; therefore, our findings cannot be extended to other age groups or ethnicities. Furthermore, the subjects enrolled can suffer from other cardiovascular diseases and comorbidities, which can act as confounding factors. Additionally, only a diabetic population with ICAS was enrolled and the cytokine serum levels changes over the follow-up period were not measured. However, our intention was to identify possible at baseline biomarkers of carotid plaque instability in this specific patient population. Finally, our sample size was small and our results need to be confirmed in a larger sample to be confirmed.

## Conclusion

The present study demonstrated that HMGB1 is an independent risk factor for carotid plaque vulnerability in an Italian population with T2DM, representing a possible molecular target to treat unstable carotid plaques and stroke prevention. However, further studies are needed to confirm the role of HMGB1 in carotid atherosclerosis and to clarify the pathophysiological mechanisms underlying plaque instability.

## Data Availability

Not applicable.
