# Correction to: Association between carotid plaque vulnerability and high mobility group box‑1 serum levels in a diabetic population

**DOI:** 10.1186/s12933-021-01376-6

**Published:** 2021-09-09

**Authors:** Federico Biscetti, Giovanni Tinelli, Maria Margherita Rando, Elisabetta Nardella, Andrea Leonardo Cecchini, Flavia Angelini, Giuseppe Straface, Marco Filipponi, Vincenzo Arena, Dario Pitocco, Antonio Gasbarrini, Massimo Massetti, Andrea Flex

**Affiliations:** 1 Fondazione Policlinico Universitario A. Gemelli IRCCS, Roma, Italy; 2 Cardiovascular Internal Medicine, Fondazione Policlinico Universitario A. Gemelli IRCCS, Catholic University School of Medicine, Largo Francesco Vito, 1, 00168 Roma, Italy; 3 Laboratory of Vascular Biology and Genetics, Università Cattolica del Sacro Cuore, Roma, Italy; 4 Vascular Surgery, Fondazione Policlinico Univer-Sitario A. Gemelli IRCCS, Roma, Italy; 5 Università Cattolica del Sacro Cuore, Roma, Italy; 6 Department of Internal Medicine, St. M. Goretti Hospital, Roma, Italy; 7 Ospedale San Giovanni Battista-ACISMOM, Roma, Italy; 8 Department of Pathology, Fondazione Policlinico Universitario A. Gemelli IRCCS, Roma, Italy; 9 Diabetology Unit, Fondazione Policlinico Universitario A. Gemelli IRCCS, Roma, Italy; 10 Department of Internal Medicine, Fondazione Policlinico Universitario A. Gemelli IRCCS, Roma, Italy; 11 Cardiovascular Surgery, Fondazione Policlinico Universitario A. Gemelli IRCCS, Roma, Italy

## Correction to: Cardiovasc Diabetol (2021) 20:114 10.1186/s12933-021-01304-8

Following publication of the original article [1], the authors noticed an error in “Methods/Study population” section. The date “June 1, 2018” which is in the “Study population” section should have been given as “June 1, 2013”. This has been corrected with this erratum.
